# Beyond Molecular Markers: The Therapeutic Significance of Mesenchymal Stem Cell Deformability in Regenerative Medicine

**DOI:** 10.3390/cells14191516

**Published:** 2025-09-28

**Authors:** Renata Szydlak

**Affiliations:** Department of Bioinformatics and Telemedicine, Faculty of Medicine, Jagiellonian University Medical College, Medyczna 7, 30-688 Krakow, Poland; renata.szydlak@uj.edu.pl

**Keywords:** mesenchymal stem cells, regenerative medicine, cell deformability, mechanotyping, homing, stemness, deformability cytometry, atomic force microscopy

## Abstract

**Highlights:**

**What are the main findings?**

- Cellular deformability is an integrative, functional biomarker of MSC quality, correlated with stemness, homing efficiency, early differentiation, and aging status.
- Real-time deformability cytometry (RT-DC) and emerging AI-based imaging predictors represent the most translatable tools for mechanotype assessment. A practical GMP-oriented framework is proposed.

**What is the implications of the main findings?**

- Incorporating deformability into ATMP quality control and sorting can enrich preparations with therapeutically potent MSC subpopulations, reduce heterogeneity, and improve clinical outcomes.
- Standardized protocols and validation, combined with multi-omics integration, can enable personalized, mechanotype-guided manufacturing of MSC therapies.

**Abstract:**

Mesenchymal stem cells (MSCs) are characterized by their unique therapeutic properties, which include the ability to differentiate, secrete paracrine factors, and migrate toward sites of tissue injury. Although classical molecular markers facilitate phenotypic characterization, they do not always reflect the true functional capacity of MSCs. This article introduces deformability, i.e., the capacity of cells to deform under mechanical forces, as a novel, integrative marker of MSC biological quality. It examines the relationship between cellular mechanical deformability and key therapeutic attributes, such as stemness, homing ability, and differentiation status. It overviews current measurement techniques, categorized by resolution, throughput, and clinical applicability. The potential applications of deformability in quality control and cell sorting for therapeutic purposes are also discussed. The article proposes that, in addition to molecular features, deformability may serve as a functional biomarker, potentially enhancing the effectiveness of MSC-based therapies.

## 1. Introduction

Mesenchymal stem/stromal cells (MSCs) are one of the most promising cell types used in regenerative medicine, immunotherapy, and tissue engineering [1,2,3]. Their therapeutic potential stems from a broad spectrum of biological functions, including the ability to differentiate into mesenchymal cells (osteoblasts, chondrocytes, adipocytes), robust paracrine activity regulating the inflammatory response and angiogenesis, and the ability to migrate and home, i.e., colonize and integrate at the site of tissue injury [4,5,6].

To ensure consistency and quality of research, the International Society for Cellular Therapy (ISCT) has defined minimum criteria to identify MSCs in vitro. According to these guidelines, MSCs should meet three main conditions: (1) adhesion to plastic under standard culture conditions; (2) expression of characteristic surface antigens (positive for CD105, CD73, and CD90 and negative for CD45, CD34, CD14 or CD11b, CD79a or CD19, and HLA-DR); and (3) the ability to differentiate into osteoblasts, adipocytes, and chondrocytes under directional culture conditions [7,8]. Although widely accepted, these criteria mainly refer to phenotypic characteristics and potential abilities of cells under laboratory conditions.

In recent years, it has become increasingly clear that such classical molecular markers, including the expression profile of CD markers and in vitro differentiation potential, are not sufficient to accurately determine the quality, functional maturity, and therapeutic efficacy of MSCs under in vivo conditions [9,10,11]. Significant discrepancies are emerging between compliance with ISCT criteria and the actual activity of cells after transplantation, including their ability to migrate, secrete cytokines, influence the host immune system, and survive at the site of injury [12,13,14]. Given this, there is a growing need to identify new, more functional and predictive indicators of the biological activity of MSCs that would better reflect their behavior and efficacy in clinical applications.

One of the most intriguing candidates for such an indicator is cell deformability, generally understood as the extent to which a cell resists or undergoes shape change when subjected to external mechanical forces. Rather than being a single property, deformability represents an integrated mechanical phenotype that emerges from the interplay of genetic, molecular, and structural determinants of the cell. It can be understood as an integrative parameter that reflects plasma membrane bending rigidity, cytoskeletal viscoelasticity, nuclear stiffness (determined by lamin A/C levels and chromatin condensation), cytoplasmic viscosity, osmotic balance, and the capacity of the whole cell to adapt its shape in confined environments [15]. In the context of MSCs, deformability may play a dual role. On the one hand, it reflects the state of the cytoskeleton and the overall “plasticity” of the cell, while, on the other hand, it may correlate with biological functions crucial for regeneration, such as migration across biological membranes, the ability to adhere to damaged tissues, and paracrine and immunomodulatory properties. Available data suggest that MSCs with higher deformability have greater homing and paracrine potential [16,17,18,19]. These cells are more flexible, allowing them to easily squeeze through narrow gaps in the endothelium and basement membrane to reach damage [20,21]. In contrast, more stiffer cells—often already in the differentiation process or after prolonged culture—are characterized by limited migration and lower secretory activity [22,23,24]. Notably, cells that have undergone a higher number of divisions are more prone to becoming stiff, reflecting replicative aging [20]. Moreover, deformability can indicate a cell’s functional age [25]. Such observations support the hypothesis that deformability may be a passive physical trait and an active, functional marker of MSCs’ regenerative capacity.

Nevertheless, deformability as a marker for MSCs faces numerous challenges. First, there are no clear, standardized protocols for measuring this parameter. Second, the available techniques vary significantly in sensitivity, throughput, and spatial resolution, making it difficult to compare results between laboratories [26,27,28]. On the one hand, high-resolution methods such as atomic force microscopy (AFM) allow for precise measurement of the nanomechanical properties of single cells under controlled conditions [20,22]. On the other hand, tools such as deformation cytometry enable real-time analysis of thousands of cells, opening the way to sorting MSC populations based on their mechanical properties [19,29,30]. At the same time, new optical strategies, such as Brillouin spectroscopy and FRET sensors, are emerging, allowing for analysis of local cell stresses without needing immobilization or labeling [31,32]. Recently, image-based deep learning models have also been introduced as a high-throughput, non-invasive method to predict MSC deformability and related functional traits directly from brightfield images, offering a scalable alternative to conventional measurement techniques [33].

This article presents arguments in favor of recognizing deformability as a functional biomarker that complements, and in some contexts may surpass, classical molecular markers in predicting the therapeutic efficacy of MSCs. It synthesizes current knowledge on the structural and molecular determinants of deformability, its relationship to stemness, migration, and differentiation status, and critically evaluates available measurement techniques regarding their precision, throughput, and translational applicability. In addition, it analyzes the integration of deformability assessment into the quality control framework for advanced therapy medicinal products (ATMPs). It discusses how this parameter could be standardized, validated, and implemented in clinical manufacturing processes. Combining knowledge from cell biology, mechanobiology, and regenerative medicine, the article aims to outline a plan for implementing mechanotyping into the next generation of personalized therapies based on MSCs.

## 2. Determinants of Cellular Deformability

Living cells and their microenvironments are inherently viscoelastic, displaying time-dependent stress relaxation and creep. Consequently, the concept of deformability should be interpreted within the framework of viscoelasticity rather than that of pure elasticity. The cytoplasm has been observed to behave as a poro-viscoelastic composite of polymer networks (e.g., actin, microtubules, intermediate filaments) and fluid phases. Similarly, the ECM demonstrates stress relaxation, whose temporal characteristics influence cytoskeletal remodeling [34,35]. Cells actively perceive and adapt to this viscoelasticity via integrin-based adhesions and mechanosensitive ion channels. These channels modulate actomyosin contractility and nuclear mechanics, thereby altering the deformability of the entire cell.

**Figure 1:**
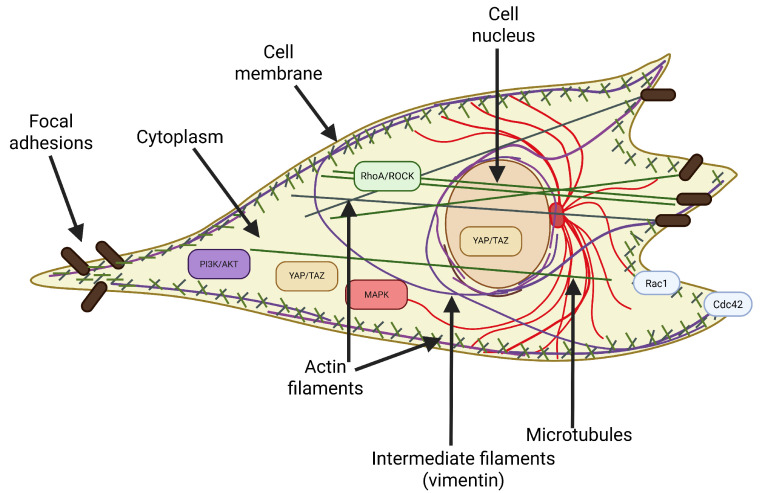
Schematic representation of a cell highlighting key structural components (F-actin cortex, microtubules, intermediate filaments, nucleus, focal adhesions) and major signaling pathways (RhoA/ROCK, Rac1, Cdc42, YAP/TAZ, MAPK, PI3K/AKT) that regulate cellular deformability.

Cell deformability is one of the fundamental mechanical properties of living cells, reflecting their ability to change shape under the influence of mechanical force [36]. It is not a static or purely physical feature, but a dynamic parameter that integrates the properties of the cytoskeleton, cell membrane, nucleus, intracellular pressure, and interactions with the microenvironment [37]. Figure 1 shows a schematic representation of a cell, emphasizing the structural components and signaling pathways that affect its deformability.

### 2.1. The Cytoskeleton as the Primary Determinant of Deformability

The cytoskeleton plays a central role in shaping the mechanical phenotype of cells [38]. It consists of three main types of filaments:

- Actin filaments (F-actin) form a dense network just below the cell membrane, called the actin cortex, responsible for the cell’s resistance to deformation under low forces. The remodeling of this network, including the formation of stress fibers, directly affects the cell’s deformability.
- Microtubules ensure the spatial stability of the cell and its resistance to compressive forces. They also play an important role in the organization of organelles and intracellular transport, and their depolymerization can indirectly affect the tension of the actin cortex.
- Intermediate filaments (mainly vimentin) are responsible for the cell’s resistance to tensile and compressive deformation. Vimentin transfers mechanical forces from the cytoplasm to the nucleus and stabilizes the cell nucleus’s position, especially during migration through narrow spaces.

Reorganization of these structures leads to significant changes in cell deformability [39,40,41,42,43].

### 2.2. Other Structural Components Affecting Deformability

Although the cytoskeleton is the main factor determining the mechanical properties of cells, other structures also significantly affect their deformability:

- The cell membrane. Its lipid composition, the presence of cholesterol, and interactions with the cytoskeleton affect its deformability and susceptibility to deformation. The lipid bilayer is intrinsically heterogeneous; cholesterol-rich “lipid-rigid” domains coexist with more compliant regions. This mosaic organization generates local variations in bending rigidity and modulates cytoskeletal anchoring. As a result, membrane composition can bias downstream cytoskeletal responses, for example, by stabilizing stress fibers in rigid lipid domains or promoting lamellipodia in more fluid regions [44,45,46]. Greater membrane fluidity may promote local deformation, but it plays a key role in the overall mechanics of the cell only in combination with the dynamic actin cortex.
- The cell nucleus. It is the largest and hardest organelle, often limiting the cell’s ability to pass through narrow spaces. The stiffness of the nucleus, which depends on the level of lamin A/C and chromatin condensation, can determine the cell’s overall deformability [47]. Mutations in LMNA (lamin A/C), reported in laminopathies, alter nuclear stiffness and compromise nuclear adaptability [48]. Such alterations may block efficient passage through confined tissue spaces, diminishing their homing efficiency and regenerative potential [49]. Pathological stiffening of the nuclear envelope also impairs chromatin organization, thereby influencing lineage commitment and accelerating features of premature senescence [50].
- Cytoplasmic viscosity determines the ease with which organelles and macromolecules move within the cytosolic space and, consequently, how the whole cell responds to deformation [51]. The cytoplasm is not a simple fluid but a crowded, viscoelastic medium whose properties are shaped by protein concentration, cytoskeletal crosslinking, and metabolic activity [52]. Increases in macromolecular crowding, aggregation of structural proteins, and oxidative stress can elevate viscosity, thereby restricting intracellular flow and reducing compliance. Conversely, active ATP-dependent processes, such as actin turnover and vesicle trafficking, can transiently fluidize the cytoplasm, enhancing its ability to deform [53].
- Osmotic pressure provides another layer of regulation of cellular deformability by directly modulating intracellular volume and hydrostatic balance. Mechano-osmotic coupling ensures that ion and water fluxes, mediated by channels and transporters such as aquaporins, Na^+^/K^+^-ATPase, and mechanosensitive ion channels (i.e., Piezo1, TRPV4), continuously adjust intracellular pressure in response to mechanical stress [54,55]. Swelling under hypo-osmotic conditions can reduce cortical tension and increase deformability, while shrinkage under hyper-osmotic stress can stiffen the cell and hinder passage through confined environments [56].
- Focal adhesions are the mechanical interface between the cytoskeleton and the substrate. Their size, number, and maturity affect the cell’s tension and ability to change shape. Strongly anchored cells have limited deformability [57].

### 2.3. Pathways Regulating Cell Deformability

Deformability is not a passive trait. It is actively regulated by a network of signaling pathways that control cytoskeletal dynamics. These pathways have been shown to coordinate cytoskeletal architecture, nuclear mechanics, adhesion dynamics, and metabolic status. The following sections offer a comprehensive overview of the canonical regulators and developmental, mechanosensory, metabolic, and epigenetic pathways that converge to determine the cellular deformability.

#### 2.3.1. Canonical Regulators (RhoA/ROCK, Rac1/Cdc42, MAPK, and PI3K/AKT)

The RhoA/ROCK pathway has been identified as the central driver of actomyosin contractility. The active form of RhoA has been shown to stimulate ROCK kinases, which in turn leads to the phosphorylation of myosin light chain (MLC) and the subsequent suppression of myosin phosphatase [58]. This results in enhanced actin stress fiber formation and increased cortical tension, which stiffens cells and reduces their deformability. High RhoA/ROCK activity in MSCs has been directly linked to osteogenic differentiation, highlighting the relationship between decreased compliance and lineage specification [59,60,61]. Pharmacological inhibition of ROCK conversely increases compliance and supports adipogenesis, illustrating how modulation of this pathway alters fate decisions [62].

In contrast, Rac1 and Cdc42 function as positive regulators of membrane protrusions. Rac1 has been shown to promote lamellipodia formation, while Cdc42 has been demonstrated to induce filopodia. These processes enhance local deformability and facilitate dynamic exploration of the extracellular environment. These activities facilitate migration through confined spaces as well as the establishment of polarity and directional movement in MSCs [63,64].

Furthermore, MAPK pathways have been demonstrated to regulate deformability. ERK activation has been shown to promote lamellipodial dynamics and proliferation. In contrast, stress-activated kinases p38 and JNK are engaged under oxidative stress, leading to cytoskeletal stiffening. The Ras/ERK axis, in particular, has been shown to support proliferation, yet it can concomitantly increase cytoskeletal rigidity, depending on the specific context. The PI3K/AKT cascade integrates growth factor signals with cytoskeletal remodeling, promoting actin polymerization, focal adhesion turnover, and nuclear positioning [65,66]. In MSCs, PI3K/AKT activity has been shown to correlate with enhanced migration and survival, while also maintaining the necessary cytoskeletal flexibility for processes such as homing and engraftment [67].

#### 2.3.2. Hippo, YAP/TAZ, and Integrators of Mechanotransduction

The Hippo pathway, mediated by MST1/2 and LATS1/2 kinases, serves as a master regulator of YAP/TAZ activity [68]. In stiff environments or under high cytoskeletal tension, Hippo kinases are inactivated, allowing YAP/TAZ to translocate into the nucleus and promote osteogenic transcriptional programs [69].

YAP and TAZ also serve as nodal points for crosstalk between other pathways, including PI3K/AKT, Wnt/β-catenin, and mTOR. Their mechanosensitive activity makes them central integrators of multiple upstream signals [70,71]. In MSCs, nuclear YAP/TAZ activity is strongly associated with osteogenic differentiation, while cytoplasmic retention is linked to maintenance of stemness and adipogenesis [72].

#### 2.3.3. Developmental and Differentiation Pathways (Wnt/β-Catenin, TGF-β/Smad, and Notch)

The Wnt/β-catenin pathway regulates both cell–cell adhesion and cytoskeletal organization [73]. Wnt ligands stabilize β-catenin, which not only acts as a transcription factor but also modulates cadherin-based adhesion complexes. Activation of Wnt signaling increases cortical stiffness and promotes osteogenesis in MSCs, while its inhibition supports adipogenic differentiation with greater deformability [74,75]. Wnt thereby acts as a biomechanical switch that connects fate commitment to mechanical state [76].

TGF-β signaling plays a dual role in MSCs. The canonical Smad pathway drives chondrogenesis and matrix deposition, while non-canonical signaling through RhoA and MAPK reorganizes the cytoskeleton [74,75]. By increasing ECM production, TGF-β indirectly alters the extracellular environment, which feeds back into cell mechanics [77]. Consequently, TGF-β signaling not only regulates differentiation but also reshapes both intrinsic and extrinsic determinants of deformability [78].

Notch signaling influences cytoskeletal stability and polarity by regulating actin organization [79]. In MSCs, Notch contributes to lineage specification, migration, and responses to inflammatory cytokines [80,81]. Notch activation has been associated with increased cytoskeletal rigidity, whereas its inhibition enhances plasticity and supports regenerative phenotypes [82].

#### 2.3.4. Mechanosensors (Integrin–FAK Signaling, Piezo/TRP Channels, and GPCRs)

Integrins are the primary transmembrane receptors sensing ECM stiffness [83]. Their engagement activates focal adhesion kinase (FAK), which regulates the assembly and turnover of focal adhesions [83]. Strong, stable adhesions stiffen the cytoskeleton, while dynamic adhesions allow for greater compliance and motility. Integrin–FAK signaling thus represents a direct mechanosensory system that tunes MSC deformability in response to substrate mechanics [84].

Mechanosensitive ion channels, including Piezo1 and TRPV4, transduce membrane stretch into intracellular calcium signals [85,86]. Calcium influx activates calpain and myosin light-chain kinase (MLCK), which regulate actin contractility [87]. In MSCs, Piezo1 is particularly important for osteogenic differentiation on stiff substrates, providing a direct mechanotransductive link between substrate stiffness and fate [86].

G-protein-coupled receptors (GPCRs), such as lysophosphatidic acid receptors, are also mechanosensitive [88,89]. GPCR activation enhances RhoA/ROCK activity, leading to increased actomyosin tension and reduced deformability [90].

#### 2.3.5. Stress and Metabolic Regulators (NF-κB, mTOR, AMPK, ROS, p53/p21, HIF-1α, and JAK/STAT)

NF-κB is classically activated by pro-inflammatory cytokines such as IL-1β and TNF-α. In MSCs, NF-κB activation induces oxidative stress, reorganizes actin filaments, and stiffens the cytoskeleton, thereby reducing deformability [91,92].

The mTOR complexes (mTORC1/2) couple nutrient availability and metabolic state with cytoskeletal organization. mTORC2, in particular, regulates actin polymerization and microtubule dynamics [93,94]. AMPK acts as an energy stress sensor and is activated under conditions of low ATP. Its activation promotes cytoskeletal remodeling toward a more compliant state, thereby protecting MSCs during metabolic stress [95].

Oxidative stress activates stress kinases such as p38 and JNK, which reinforce cytoskeletal stiffening and impair regenerative potential [96,97]. The p53/p21 axis, a hallmark of senescence, induces actin stress fibers and nuclear rigidity, further reducing deformability [98]. Hypoxia stabilizes HIF-1α, which reprograms metabolism toward glycolysis and enhances cytoskeletal plasticity [99]. Hypoxia-conditioned MSCs often display greater deformability and improved homing capacity [100].

Finally, JAK/STAT signaling, activated by cytokines such as IL-6 and IFN-γ, modulates immunomodulatory activity as well as cytoskeletal organization [101,102]. Through this pathway, inflammatory cues can either increase stiffness or enhance motility, depending on the cellular context.

#### 2.3.6. Epigenetic Regulation and Nuclear Mechanics

Epigenetic remodeling and nuclear architecture are major determinants of cellular deformability. Histone modifications and chromatin condensation directly alter nuclear stiffness, with open chromatin states being more compliant [103,104]. Lamin proteins connect the nuclear envelope to the cytoskeleton, and mutations in lamin A/C lead to altered nuclear deformability and whole-cell mechanics [105,106].

### 2.4. Matrix Elasticity as a Determinant of Deformability

Among the external regulators of cellular mechanics, matrix elasticity plays a particularly critical role [35,47,58]. The pioneering studies of Engler and Discher demonstrated that naive MSCs are exquisitely sensitive to substrate stiffness. Soft matrices mimicking brain promote neurogenesis, intermediate stiffness resembling muscle favors myogenesis, and rigid matrices characteristic of osteoid drive osteogenesis. These findings established that matrix elasticity not only guides lineage specification but also feeds back onto the cytoskeleton and nuclear mechanics, thereby modulating cell deformability itself [35,47,58]. Importantly, mechanotransduction through integrins, focal adhesions, and actomyosin contractility links ECM stiffness to intracellular tension and nuclear remodeling, which together define how cells deform under stress. MSCs on stress-relaxing substrates display faster spreading and enhanced adaptation of cortical architecture compared with purely elastic substrates, resulting in measurable shifts in deformability distributions. Mechanoperception occurs via integrins, glycocalyx-mediated load filtering, and stretch-activated channels; together, these pathways “tune” deformability as a function of environmental timescales [34].

## 3. Deformability of MSCs and Their Regenerative Competence

The deformability of MSCs is not a static or purely mechanical feature. It results from a dynamic balance between the cytoskeletal structure, cell membrane properties, the metabolic state of the cell, and interactions with the microenvironment. In this context, the deformability of MSCs is a complex parameter that integrates many aspects of their functional biology and reflects their ability to perform therapeutic functions.

### 3.1. Deformability Reflects Stemness and Functional Immaturity

The deformability of MSCs is a highly integrated indicator of their functional status and degree of immaturity. Cells with higher deformability typically represent earlier stages of differentiation, retaining proliferative potential and a more plastic molecular phenotype. In contrast, decreased deformability is generally associated with reduced regenerative capacity and loss of stemness [22,107].

Studies using AFM and optical stretching have shown that MSCs subjected to long-term in vitro culture undergo progressive mechanical changes, and their deformability decreases proportionally to the number of population doublings [22]. Maloney et al. and Szydlak et al. noted that cells from higher passages are stiffer and divide more slowly, indicating a decrease in their replicative and regenerative capacity [20,22]. After just a dozen or so passages, MSCs become noticeably stiffer, as reflected by a significant increase in Young’s modulus, even if they still express phenotypic markers consistent with ISCT criteria. This phenomenon is associated with the reorganization of actin filaments, increased cortical tension, and the activation of p53/p21 pathways [25]. Aging cells exhibit reduced proliferative capacity, a weaker paracrine response, and lower therapeutic efficacy in vivo [25].

Importantly, while associations between reduced deformability and hallmarks of cellular aging (e.g., p53/p21 activation, cytoskeletal remodeling) are well-documented, the causal directionality remains under investigation [108,109]. Deformability should therefore be regarded as a sensitive correlate, rather than a definitive biomarker, of early functional decline, and future studies are needed to clarify its mechanistic role in the aging process.

### 3.2. Deformability Is a Determinant of Migration and Homing

The deformability of MSCs is not only a morphological feature, but also a functional one, closely related to their ability to migrate, penetrate the endothelium, avoid mechanical arrest, and efficiently home to target tissues [110]. For a long time, it was assumed that MSCs’ migration is mainly based on ligand–receptor interactions. However, a growing body of data indicates that mechanical properties, such as deformability, are an equally important determinant of their biodistribution and therapeutic efficacy [19,21,111].

MSCs cultured in vitro reach a diameter of 15–30 µm, which far exceeds the lumen of capillaries, especially in the lungs (10–15 µm). As a result, most intravenously administered cells are mechanically arrested in the pulmonary capillaries (a phenomenon known as the “first-pass effect”) [107,112,113,114]. While it may serve some protective function, it effectively restricts cell access to tissues affected by damage or inflammation.

In this context, deformability gains importance. Cells that are more flexible can change shape and squeeze through microvessels despite their seemingly unfavorable geometry [112]. Moreover, deformability correlates with the rate of passing through microchannel constrictions, as observed for MSCs cultured in three-dimensional aggregates [115]. These cells were significantly smaller, softer, and more deformable, and their presence in organ microcirculation (liver, heart, kidney) after intravenous injection was significantly higher than that of classically cultured plastic MSCs [116,117].

Successful migration of MSCs across the endothelial barrier relies on their biomechanical adaptability, which is dynamically tuned by chemotactic and adhesion pathways such as SDF-1/CXCR4, HGF/c-Met, and PDGF signaling in cooperation with integrins. These cues orchestrate actin cytoskeleton remodeling, regulate myosin-II-driven contractility, and influence nuclear mechanics, thereby modulating whole-cell deformability in real time [110,118]. For instance, SDF-1/CXCR4 activation enhances actin remodeling via PI3K/AKT and Rho GTPases, thereby lowering the mechanical threshold for transendothelial migration [119,120,121]. HGF/c-Met signaling promotes migration through MAPK- and FAK-dependent pathways that soften the cytoskeleton and facilitate cellular elongation [122]. Similarly, PDGF pathways interacting with integrins coordinate focal adhesion dynamics and intracellular tension, helping MSCs adapt their deformability to vascular shear forces [120]. In an in vitro model, both IL-1β and IL-6 were shown to induce transendothelial migration and invasion across the basement membrane [20]. However, only pre-incubation of cells with IL-1β further enhanced their migratory capacity, which may indicate the existence of a regulatory mechanism related to activation of the p38/MAPK pathway [20]. Importantly, AFM measurements showed that a subpopulation of cells with higher deformability, i.e., a lower Young’s modulus, selectively passed through the endothelial barrier and basement membrane [20]. Migration and invasion were thus not only the result of chemotaxis, but also the mechanical properties of the cells, and increased deformability proved to be a key factor in enabling effective penetration through physical barriers. Most importantly, however, the subpopulations of cells that passed through the endothelium and extracellular matrix had a significantly lower Young’s modulus (an ~40% decrease), indicating increased deformability [20]. Studies using AFM showed a clear shift in deformability distribution toward lower values for migrating cells, confirming that deformability plays a selective role in the migration mechanisms of MSCs.

The ability to deform can be used as a functional biomarker to assess the homing and migration potential of MSCs. In mesensphere MSC models, morphorheological parameters such as a low Young’s modulus and rapid overcoming of microchannels correlated with a more uniform distribution of cells outside the lung [115]. In models of WJ-MSCs, the change in deformability in response to IL-1β occurred even without apparent changes in the morphology of the F-actin cytoskeleton, suggesting the existence of more subtle, signal-regulated reorganization mechanisms [20].

The findings suggest that manipulating the deformability of MSCs, such as through appropriate culture techniques (e.g., 3D), cytokine preconditioning, or biomechanical selection, can significantly enhance their therapeutic efficacy. In systemic therapies, such as treating inflammatory diseases, myocardial infarction, or hematopoietic cell transplantation, deformability may be as important as classical phenotypic markers.

From the perspective of designing cellular therapies, the biomechanical properties of MSCs should be considered an important parameter that can be measured, modified, and used to increase the efficiency of cell homing to the target tissue.

### 3.3. Deformability and Differentiation Status of MSCs

Differentiation of MSCs is accompanied by profound changes in the organization of their cytoskeleton, mechanical phenotype, and viscoelastic properties [123]. These changes directly affect the deformability of the cells and indicate a transition from a multipotent, regenerative state to a cell-lineage-determined phenotype. Accordingly, deformability serves not only as a reflection of stemness, but also as a sensitive marker of differentiation progression [20,22].

In the early stages of differentiation, MSCs gradually transition from a state of mechanical susceptibility to one characterized by decreased deformability and plasticity. This transition is closely linked to cytoskeletal remodeling, including formation of actin stress fibers, increased maturation of focal adhesions, and reorganization of intermediate filaments. These structural changes increase cortical tension and Young’s modulus values, as consistently observed in the osteogenic, adipogenic, and cartilaginous differentiation pathways [124,125,126].

Osteogenic differentiation of MSCs has been shown to require high cytoskeletal tension. It is promoted by activation of the RhoA/ROCK signaling pathway, which is associated with decreased cellular deformability and a spread cell morphology [127]. Differentiating osteoblasts show increased actin binding and stiffening of nuclei through upregulation of lamina A/C [49,124]. In contrast, adipogenic differentiation leads to a moderate increase in deformability compared with osteogenesis, especially in the early stages. However, as lipid accumulation progresses, cells become increasingly round and less mechanically stable due to cytoskeletal breakdown [128,129]. Studies indicate that, in 3D tissue with a soft matrix, chondrogenesis is promoted regardless of the complex organization of the cytoskeleton, while more rigid substrates affect differentiation by mechanotransduction [130].

Importantly, the decrease in deformability during differentiation occurs even without clear changes in surface marker expression, highlighting the potential of deformability as an early, functional indicator of cell lineage commitment [22]. In addition, pharmacological inhibition of RhoA/ROCK signaling has been shown to reverse cytoskeletal stiffening and restore partial deformability, further supporting a cause-and-effect relationship between cytoskeletal tension and differentiation status [24]. Moreover, mechanotransduction signals transmitted by YAP/TAZ pathways seamlessly integrate biochemical and biomechanical signals, and nuclear localization of YAP/TAZ on stiffer substrates promotes osteogenesis. In comparison, their cytoplasmic retention on softer substrates promotes adipogenesis [131,132].

These findings suggest that cell deformability reflects and actively modulates fate decisions through mechanosensitive transcriptional programs. From a therapeutic perspective, the mechanosensitive phenotype of MSCs serves as a valuable indicator of their differentiation status and regenerative potential.

## 4. Measurement Techniques: From Biophysical Tools to Translational Applications

The measurement of cell deformability, long marginalized as a niche topic in biophysics, is now becoming an important element of modern cell assessment. A cell’s mechanical properties, and in particular its ability to deform, can serve as an integrated functional biomarker. In the context of MSCs to be used in clinical practice, the question arises as to not only whether we can measure deformability but also how we can do so reliably and efficiently enough to make it a practical selection and standardization criterion.

The most important groups of measurement techniques are discussed below and divided according to their sensitivity, throughput, and translational applicability in order to identify tools with real potential for implementation in regenerative medicine. Table 1 summarizes the key parameters of available techniques, including their measurement scale, throughput, invasiveness, GMP applicability, and major advantages and limitations, to facilitate the comparison of techniques.

### 4.1. High-Resolution, Low-Throughput Techniques

Techniques such as AFM and micropipette aspiration offer exceptional precision in assessing the mechanical properties of individual cells [57]. AFM enables mapping of deformability distribution at the subcellular level and has been shown to distinguish MSCs in different stages of differentiation and aging [125,133]. Similarly, micropipette aspiration allows the viscoelasticity of a cell to be assessed by applying a known pressure and observing the length of its deformation in a capillary [134].

These methods provide quantitative, mechanistic insight into cell deformability. However, their translational application is limited by their low throughput, long measurement times, and technical complexity, making them unsuitable for routine batch screening in GMP production.

### 4.2. Medium-Throughput Techniques

Optical stretching uses focused laser beams to deform cells suspended in fluid, allowing for measurement of their mechanical response without physical contact with the substrate or labeling [135,136]. This approach offers higher throughput than AFM. It has been successfully applied to compare the mechanical phenotypes of MSCs from different culture conditions and passages [107]. However, limitations such as specialized optical equipment and moderate throughput hinder its integration into large-scale manufacturing processes.

### 4.3. High-Throughput Techniques

**Figure 2:**
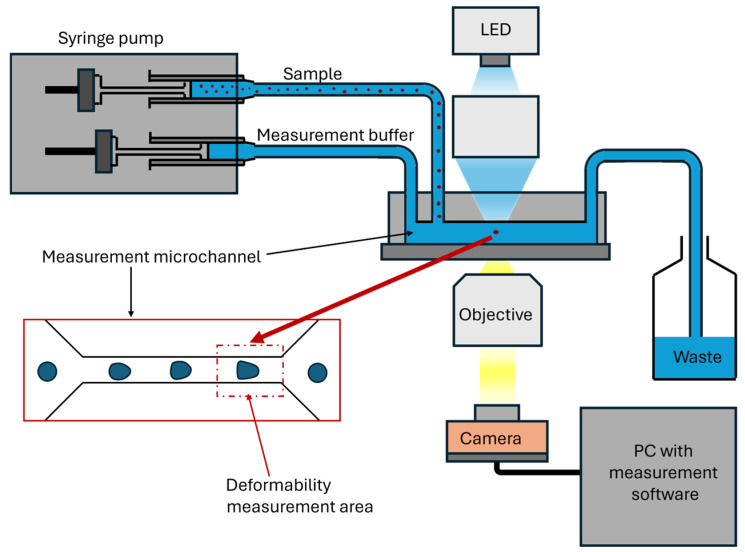
Real-time deformability cytometry (RT-DC)—instrument and measurement principle. Syringe pumps deliver the sample and measurement buffer, which hydrodynamically focuses cells in the measurement microchannel. In the designated deformability measurement area, cells are transiently deformed under laminar flow. LED illumination and the objective project the image onto a high-speed camera. The PC performs real-time segmentation and contour extraction and computes cell size and deformability.

A breakthrough for clinical applications is real-time deformability cytometry (RT-DC), which measures the deformability of thousands of cells per second without needing labeling, immobilization, or a change in culture conditions [26,137]. Cells flow through a microfluidic channel, where they undergo hydrodynamic compression, and a high-speed camera records their degree of deformation (Figure 2). RT-DC enables rapid mechanical profiling of MSC populations and, thanks to the development of integrated sorters, enables the selection of subpopulations with higher therapeutic potential.

RT-DC is currently the most promising translational technique for mechanical phenotyping of MSCs, as it combines speed, repeatability, and compatibility with GMP conditions.

### 4.4. Next-Generation Techniques

Emerging approaches aim to predict deformability without direct mechanical measurement. These include deep learning models and artificial intelligence trained to infer mechanical phenotypes from brightfield or phase-contrast images [33]. Once the model has learned to recognize morphological features associated with the mechanical phenotype, it can be used to predict these parameters in new, previously unexamined cells without the need for any physical mechanical testing.

Using neural networks to extract correlations between images and mechanical parameters may enable non-invasive and low-cost monitoring of MSCs’ properties without physical measurements based solely on standard microscopic images.

## 5. Translational Applications

### 5.1. Deformability as a Quality Control Criterion

In producing advanced therapy medicinal products (ATMPs), rapid, label-free, and non-destructive methods are required to assess the quality of large cell populations without destroying them. Deformability, as a parameter associated with regeneration potential, homing efficiency, and resistance to mechanical stress, can serve as a functional biomarker complementing classical molecular indicators.

Using RT-DC to monitor the deformability of MSC batches may enable more precise control of their biological quality [137,138,139]. This technique excludes subpopulations with reduced deformability, which is often associated with replicative senescence or initiated differentiation. At the same time, selecting more flexible subpopulations with better migration and homing abilities is possible. In addition, RT-DC can be used to assess the impact of various cell processing procedures, such as trypsinization, freezing, or exposure to cytokines, on their mechanical phenotype. As a result, deformability can serve as a functional indicator for the early detection of adverse changes in preparations intended for cell therapy. This approach is in line with Good Manufacturing Practice (GMP) requirements and can support the qualification process of therapeutic batches prior to patient administration.

### 5.2. Selection of Subpopulations with Increased Therapeutic Efficacy

Within heterogeneous MSC preparations, there may be subpopulations with significantly different deformability and therapeutic efficacy. The integration of RT-DC with a sorter enables the physical separation of cells with the desired mechanotype, e.g., deformable cells with a low Young’s modulus, correlating with the potential for migration, avoidance of the “first-pass effect”, and effective homing to target tissues [140,141]. Such strategies are beneficial in systemic therapies, for example, treatment of inflammatory conditions (e.g., Crohn’s disease), heart regeneration, and hematopoietic transplants, where rapid cell delivery to the bone marrow is important.

### 5.3. Integration of Deformability with Multiomics Approaches

In order to more fully characterize the functionality of MSCs, deformability can be integrated with transcriptomic, epigenetic, and metabolomic data [142]. In recent years, models have emerged that combine gene expression profiles with cell deformability and their response to the microbiological environment or cytokines [143,144,145]. This allows cells to be classified in terms of quality and predicts their behavior in vivo. In the future, deformability may be part of a complex algorithm for predicting cell survival after transplantation, predicting their ability to modulate the immune system, and assessing susceptibility to spontaneous differentiation.

### 5.4. Clinical and Regulatory Perspectives

For deformability to be implemented in clinical practice as a parameter for assessing MSC quality, several key conditions must be met. First and foremost, it is necessary to standardize measurement protocols, including aspects such as flow conditions, temperature, and the type of buffer used. At the same time, it is necessary to validate the correlation between deformability and therapeutic efficacy in preclinical and clinical studies. Only on this basis will it be possible to further implement this parameter in evaluating cell batches used in therapies. Ultimately, recognizing deformability as an important indicator of cell therapy quality also requires its formal acceptance by regulatory authorities such as the EMA and FDA as a parameter supporting ATMP quality control. In this context, MSC mechanotyping can be considered a research tool and a decision-making component in approving a product for clinical use.

### 5.5. Practical Framework for Implementing MSC Deformability Assessment in ATMP Manufacturing

To facilitate the integration of deformability measurements into GMP-compliant manufacturing processes, the following stepwise decision-making framework can be applied:

1. Initial cell harvesting and isolation: Assess the initial deformability of freshly isolated MSCs to establish donor- and tissue-specific reference values.
2. Expansion phase monitoring: Perform periodic assessments of MSCs’ deformability during culture using RT-DC to detect early mechanical changes associated with aging or unintended differentiation.
3. Quality control before clinical introduction: Determine the final deformability profile at the batch level before product launch and verify and exclude subpopulations with increased stiffness, indicating reduced self-guidance or regeneration capacity.
4. Post-thaw verification (if cryopreserved): Assess deformability after thawing to confirm recovery of the mechanical phenotype prior to administration.

This framework enables proactive identification of suboptimal mechanotypes, ensures batch consistency, and supports regulatory documentation. Future workflows may automate this process through closed-system RT-DC sorters, integrating deformability-based selection directly into production lines.

## 6. Challenges and Future Directions

Despite compelling evidence linking MSCs’ deformability to their regenerative capacity, several significant barriers hinder their adoption as a primary functional biomarker in clinical practice.

One of the key challenges is the lack of standardized measurement protocols. Current methods, ranging from high-resolution but low-throughput techniques such as AFM to rapid population-level tests such as RT-DC, vary significantly regarding sensitivity, mechanical loading regimes, environmental conditions, and data analysis procedures. This heterogeneity limits the comparability of studies and hinders meta-analytical validation. Although a standardized measurement protocol covering culture, calibration, measurements, and data analysis has been developed for AFM, which has allowed for consistent Young’s modulus values to be obtained in multiple laboratories [28], the lack of analogous standards for RT-DC still poses a significant barrier to its use as a routine quality control tool in GMP-compliant MSC production.

Another important challenge is determining when to assess MSCs’ deformability and under what conditions. MSCs exhibit different mechanical properties in vitro (e.g., on a stiff plastic substrate, where they form numerous focal adhesions), in ex vivo 3D systems (where adhesion is limited), and in vivo, where they interact with a dynamic, viscoelastic microenvironment. The lack of standardization of these conditions makes it difficult to compare results between laboratories and draw clinical conclusions. According to GMP recommendations, standardization encompasses measurement methods, culture conditions, number of passages, tissue source, and method of cell preparation. These differences have direct practical significance. MSCs stored as cryobanked preparations and thawed immediately before administration may differ in mechanotype from freshly cultured cells. This difference affects their ability to penetrate endothelial barriers and home. Therefore, future work should clearly define standard measurement points for deformability (e.g., before cryopreservation, after thawing, and during expansion under 2D and 3D conditions) and develop assessment systems that better reflect physiological conditions. Only such an approach will enable us to link in vitro results with the actual clinical efficacy of MSC therapies.

An additional challenge is the biological heterogeneity of MSC populations, both between donors and within a single culture [146]. Factors such as donor age, tissue source (e.g., bone marrow, fat, Wharton’s jelly), passage number, and culture conditions (2D vs. 3D, substrate stiffness) affect cell mechanics [22,29]. The mechanotype of MSCs changes in response to inflammation, mechanical stress, and cytokine exposure [20]. Although biologically beneficial, this plasticity poses a challenge in quality control. At what point in production should deformability be assessed, and how stable does this parameter remain during transport, storage, or regeneration after thawing?

In vitro studies indicate that greater deformability correlates with better migration and homing, but there is no clear evidence of causality concerning clinical efficacy [20,115]. For deformability to gain status as a criterion in ATMP qualification, the predictive value of the mechanotype must be confirmed in preclinical and clinical studies. Studies are needed in which MSC preparations are categorized according to mechanotype and monitored for regenerative outcomes in patients.

Although deformability offers valuable functional readouts, its predictive power can be maximized by integrating it with complementary omics datasets such as transcriptomics, epigenomics, proteomics, and metabolomics. Multidimensional profiling can generate complex potential indicators, reflecting both mechanical performance and molecular signatures, enabling more precise selection of therapeutic populations.

New approaches, including AI-based morphomechanical prediction, label-free optical nanomechanics, and integrated mechanomic methods, have the potential to make deformability assessment more accessible, automated, and scalable. Combining RT-DC with GMP sorters in a closed system could enable real-time enrichment of therapeutic cell batches to obtain optimal mechanotypes without compromising sterility and viability.

The inclusion of deformability as a key functional biomarker represents a paradigm shift from static phenotypic characterization toward a dynamic, mechanism-based assessment of MSCs’ potential. Combining biophysics, cell biology, and biomedical engineering can improve reproducibility in production, increase the precision of therapeutic cell selection, and ultimately enhance the clinical efficacy of MSC-based interventions. In the long term, combining deformability with multiomic profiles and predictive modeling may form the basis for a new generation of personalized regenerative therapies.

## 7. Conclusions

Deformability has emerged as a robust functional biomarker that records the integrated mechanical, molecular, and metabolic state of MSCs. Unlike static phenotypic markers, it reflects dynamic properties directly related to stemness, migratory competence, and differentiation status, which are key features for the therapeutic efficacy of MSCs in regenerative medicine. Advances in high-throughput, label-free measurement techniques, particularly real-time deformability cytometry, pave the way for the routine implementation of mechanotyping in GMP-compliant medicine.

Including deformability in quality control frameworks offers a practical path to increasing the precision of MSC-based therapies by enabling the selection of cell populations with higher homing potential and regenerative efficacy. However, this change requires standardized measurement protocols, robust preclinical and clinical validation, and formal recognition by regulatory authorities.

Integrating deformability with multiomics and AI-based morphomechanical analytics can transform MSC characterization from descriptive profiling to predictive, patient-tailored therapeutic approaches. By combining biophysical phenotyping with system-level data, deformability assessment can serve as a quality gatekeeper and a roadmap for the next generation of personalized regeneration techniques.

## Figures and Tables

**Table 1:** Comparative overview of techniques for measuring the deformability of cells, including their measurement scale, throughput, invasiveness, GMP applicability, and key advantages and limitations.

Technique	Scale	Throughput	Invasiveness	GMP Applicability	Advantages	Limitations
Atomic force microscopy	Local and global *	Very low (<30 cells/h)	Contact-based, label-free	Low	High precision, subcellular mapping, detects subtle changes	Time-consuming, requires a skilled operator, low throughput, requires adherent cells
Micropipette aspiration	Local and global	Very low (<10 cells/h)	Contact-based, label-free	Low	Simple principle, direct viscoelastic measurement	Manual operation, limited scalability, low throughput, requires adherent cells
Optical stretching	Global	Moderate (<1000 cells/h)	Contact-free, label-free	Medium	Non-contact, suitable for suspended cells	Requires specialized optics, moderate throughput
Real-Time Deformability Cytometry	Global	Very high (<10,000 cells/s)	Hydrodynamic contact, label-free	High	High speed, label-free, suitable for suspended cells, enables sorting	Requires microfluidic setup, lacks universal standards
AI-based imaging prediction	Local and global	Very high (imaging-limited)	Non-invasive, label-free	Potentially high	Non-invasive, scalable, low cost after training, no physical manipulation	Requires a large annotated dataset, indirect measurement

* Wedged cantilever mode enables global mechanical mapping in AFM.

## Data Availability

No new data were created or analyzed for this article.

## Abbreviations

The following abbreviations are used in this manuscript:

AFM	Atomic Force Microscopy
AI	Artificial Intelligence
ATMP	Advanced Therapy Medicinal Product
EMA	European Medicines Agency
FAK	Focal adhesion kinase
FDA	Food and Drug Administration
FRET	Forster Resonance Energy Transfer
GMP	Good Manufacturing Practice
GPCR	G-protein coupled receptors
IL-1β	Interleukin 1 β
IL-6	Interleukin 6
ITSC	International Society for Cellular Therapy
MAPK	Mitogen-Activated Protein Kinase
MLC	Myosin light chain
MSC	Mesenchymal Stem Cell
RT-DC	Real-Time Deformability Cytometry
TAZ	Transcriptional Coactivator with PDZ-binding motif
YAP	Yes-Associated Protein
